# Among-Strain Variation in Resistance of *Paramecium caudatum* to the Endonuclear Parasite *Holospora undulata*: Geographic and Lineage-Specific Patterns

**DOI:** 10.3389/fmicb.2020.603046

**Published:** 2020-12-14

**Authors:** Jared Weiler, Giacomo Zilio, Nathalie Zeballos, Louise Nørgaard, Winiffer D. Conce Alberto, Sascha Krenek, Oliver Kaltz, Lydia Bright

**Affiliations:** ^1^Department of Biology, State University of New York, College at New Paltz, New Paltz, NY, United States; ^2^ISEM, University of Montpellier, CNRS, EPHE, IRD, Montpellier, France; ^3^School of Biological Sciences and Centre for Geometric Biology, Monash University, Melbourne, VIC, Australia; ^4^Division of Infectious Diseases, Department of Medicine, Weill Cornell Medicine, New York, NY, United States; ^5^Institute of Hydrobiology, Technische Universität Dresden, Dresden, Germany

**Keywords:** genotype, phenotype, heritability, symbiont, repeatability, cytochrome oxidase I, haplogroup

## Abstract

Resistance is a key determinant in interactions between hosts and their parasites. Understanding the amount and distribution of variation in this trait between strains can provide insights into (co)evolutionary processes and their potential to shape patterns of diversity in natural populations. Using controlled inoculation in experimental mass cultures, we investigated the quantitative variation in resistance to the bacterial parasite *Holospora undulata* across a worldwide collection of strains of its ciliate host *Paramecium caudatum*. We combined the observed variation with available information on the phylogeny and biogeography of the strains. We found substantial variation in resistance among strains, with upper-bound values of broad-sense heritability >0.5 (intraclass correlation coefficients). Strain estimates of resistance were repeatable between laboratories and ranged from total resistance to near-complete susceptibility. Early (1 week post inoculation) measurements provided higher estimates of resistance heritability than did later measurements (2–3 weeks), possibly due to diverging epidemiological dynamics in replicate cultures of the same strains. Genetic distance (based on a neutral marker) was positively correlated with the difference in resistance phenotype between strains (*r* = 0.45), essentially reflecting differences between highly divergent clades (haplogroups) within the host species. Haplogroup A strains, mostly European, were less resistant to the parasite (49% infection prevalence) than non-European haplogroup B strains (28%). At a smaller geographical scale (within Europe), strains that are geographically closer to the parasite origin (Southern Germany) were more susceptible to infection than those from further away. These patterns are consistent with a picture of local parasite adaptation. Our study demonstrates ample natural variation in resistance on which selection can act and hints at symbiont adaptation producing signatures in geographic and lineage-specific patterns of resistance in this model system.

## Introduction

Evolutionary interactions between hosts and their symbionts can shape patterns of genetic diversity and adaptation in natural communities (Kaltz and Shykoff, 1998; Howells et al., 2013; Caldera et al., 2019). For this diversification to occur there must be heritable variation in the traits determining the “compatibility” between a host and a symbiont, from resistance and infectivity to more complex life-history or developmental traits (Law and Dieckmann, 1998; Dale and Moran, 2006; Chomicki et al., 2020). In many systems, this variation can be assessed in controlled infection experiments, using different combinations of host and symbiont genotypes (Carius et al., 2001; Kaltz and Shykoff, 2002; Cayetano and Vorburger, 2013). Such experiments not only inform on the present evolutionary potential of the interacting players, but can also reveal patterns shaped by their (co)evolutionary past, such as local adaptation (Greischar and Koskella, 2007; Hoeksema and Forde, 2008), lineage or species specificity (Summerer et al., 2007; Chappell and Rausher, 2016) and symbiont life styles (Degnan and Moran, 2008; Sachs et al., 2011).

The ciliated protist *Paramecium* spp. is a useful model organism for experimental investigations into diverse associations with endosymbiotic bacteria that represent the full continuum of endosymbiotic interactions: from facultative to obligate, and from horizontal to mixed to vertical transmission (Preer et al., 1974; Fokin and Görtz, 2009; Fujishima, 2009; Görtz and Fokin, 2009; Goertz, 2010; Fujishima and Kodama, 2012; Plotnikov et al., 2019). The possession of these endosymbiotic partners is likely the result of *Paramecium*’s bacterivorous nature, whereby some of its prey have evolved means to evade digestion and to take up residence in the cytoplasm of the cell, and also in the host nuclei (Goertz, 2010; Boscaro et al., 2013; Schulz and Horn, 2015; Garushyants et al., 2018; Potekhin et al., 2018; Munoz-Gomez et al., 2019). For many of these associations, the biology and evolution are still poorly understood. Among the notable exceptions is the genus Holospora, a group of Alphaproteobacteria distantly related to other endosymbionts, such as Rickettsia or Wolbachia (Szokoli et al., 2016). Following the discovery of *Holospora* bacteria in the 19th century (Hafkine, 1890), there has been extensive research on their morphology, infection life cycle, and taxonomic relationships [for a historical overview, see Fokin and Görtz (2009)]. More recently, the *Paramecium-Holospora* system has served as a model to study epidemiology and evolution in experimental microcosms (Lohse et al., 2006; Nidelet et al., 2009; Duncan et al., 2011a, 2015, 2018; Castelli et al., 2015; Dusi et al., 2015; Nørgaard et al., 2020; Zilio et al., 2020a, b).

*Holospora* are highly species-specific endonuclear symbionts of *Paramecium* spp. with each species specifically infecting either the somatic macronucleus (MAC) or the germline micronucleus (MIC) of the host cell (Fokin and Görtz, 2009; Fujishima, 2009). Typically, *Holospora* species infect one single host species, even though temporary infection of other “non-host” species is possible (Fujishima and Fujita, 1985; Fokin et al., 2005). Only the phylogenetically more distant *Holospora caryophila* (now *Ca. Preeria caryophila*) is known to stably occur on multiple host species (Potekhin et al., 2018). Furthermore, the interaction appears to be obligate for the bacterium, based on two lines of evidence; first, the life cycle of the bacterium inside of host cells (Fokin, 1998; Fokin and Görtz, 2009) and the second, that recent comparative analysis of several *Holospora* genomes indicates that most amino acid synthesis pathways are absent, along with other biosynthetic pathways (Garushyants et al., 2018) and therefore replication must only be possible inside the host cell. Reproductive forms of the bacteria (RFs) can be vertically transmitted from mother to daughter nuclei during host cell division (Fujishima, 2009; Magalon et al., 2010), whereas horizontal transmission occurs when infectious forms (IFs) are released during cell division or upon host death and then ingested by new hosts while feeding. *Holospora* symbionts show typical parasite features: they disrupt sexual processes (Görtz and Fujishima, 1983; Fokin, 1998) and reduce asexual division, cell survival, and motility (Restif and Kaltz, 2006; Duncan et al., 2010; Bella et al., 2016; Nørgaard et al., 2020; Zilio et al., 2020b). All *Holospora* species possess the molecular pathways to use nucleotides as an energy source (Garushyants et al., 2018), which provides a possible physiological explanation for the observed negative effects on the host phenotype. Consistent with response to a parasite, several host resistance mechanisms have been described (Rautian et al., 1993; Skoblo et al., 1996; Fokin and Skovorodkin, 1997; Fokin et al., 2003a,b; Goertz, 2010); they include reduced uptake of IFs (Fels et al., 2008), inability (of the bacteria) to enter the nucleus (Rautian et al., 1993; Fokin et al., 2003b), and/or lysis of the bacteria after establishment in the nucleus (Rautian et al., 1993; Fokin and Skovorodkin, 1997; Skovorodkin et al., 2001; Fokin et al., 2005; Goertz, 2010).

Little is known about the ecological genetics of *Paramecium-Holospora* interactions in natural populations. *Holospora* have been found at low infection prevalences in temperate or elevated locations around the globe (Fokin et al., 1993, 2006, 2004; Hori and Fujishima, 2003; Serra et al., 2016), but can occasionally cause local epidemics (Fokin and Görtz, 2009), which might give a strong selective advantage to resistant variants over susceptible ones. Previous cross-inoculation studies demonstrated the existence of such natural variation, showing that different strains of *Paramecium caudatum* (and several other species) have different qualitative infection phenotypes, with some strains appearing universally susceptible to infection with *Holospora*, while others are more difficult to infect or even entirely resistant (Fujishima and Fujita, 1985; Rautian et al., 1993, 1996; Skoblo et al., 1996; Fokin et al., 2003b; Potekhin et al., 2018). Previous studies have also compared resistance across independent mating groups or “syngens.” Syngens have long been known for *P. caudatum* (Gilman, 1941), and although reproductive isolation may not always be complete (Tsukii and Hiwatashi, 1983; Johri et al., 2017), genetic analyses identified various clades in *P. caudatum* that can be considered as independent evolutionary units (IEUs) (Barth et al., 2006; Hori et al., 2006; Johri et al., 2017; De Souza et al., 2020). These clades diverged up to 20 MYA ago (De Souza et al., 2020) and potentially represent “cryptic species” that are morphologically identical, but genetically isolated. Given the strong species-specificity of *Holospora*, such cryptic species complexes may represent an important structural component of resistance variation in natural populations. Indeed, Fujishima and Fujita (1985) found that *Holospora obtusa* was only able to infect strains from 5 out of 6 of the *P. caudatum* syngens studied, whereas an extensive study on *Holospora undulata* revealed successful infection of 84 of 92 *P. caudatum* strains and all 10 syngens tested (Skoblo et al., 1996). In yet another system, Rautian et al. (1993) reported evidence of strong syngen-level specificity of strains from *Holospora acuminata* in *Paramecium bursaria*, and this across a wide range of geographic origins.

However, while most of the classic studies have investigated the qualitative aspects of resistance, it clearly is a continuous trait: inoculation of experimental cultures typically results in higher or lower proportions of infected individuals (Lohse et al., 2006; Fels et al., 2008). Whether this reflects a polygenic basis of the trait or is simply due to the probabilistic nature of the infection process is unclear, but obviously continuous trait variation may produce very different epidemiological or evolutionary dynamics than qualitative variation (Duncan et al., 2011b).

Our goal in this study was to provide a rigorous analysis of the amount and distribution of quantitative among-strain variation in resistance in the *Paramecium-Holospora* system. We performed a resistance assay testing 30 *P. caudatum* strains from a worldwide collection against the *H. undulata* reference strain (Dohra et al., 2013). The strains derive from independent wild isolates presumably with different genetic backgrounds and potentially very different evolutionary histories. It is therefore conceivable that the germline micronuclei of these strains harbor divergent genomes (in this sense, strains can be referred to as “genotypes”). For our purposes, all strains were propagated as clonal replicate cultures and then challenged with a parasite inoculum. We assume that the observed phenotypic variation in resistance among the strains results from differential gene expression in the somatic macronucleus. Using basic statistical tools, we quantified this amount of among-strain variation (broad-sense heritability) and compared this variation between highly divergent clades, mainly from two haplogroups (Europe vs. rest of world). We further provide a first set of tentative analyses linking phylogenetic distances (based on a neutral genetic marker), geographic distances and trait variation among the strains.

## Materials and Methods

### *Paramecium caudatum* Strains and Culturing

The *P. caudatum* strains were taken from a collection with worldwide distribution (Supplementary Table 1) at the Institute of Hydrobiology (TU Dresden, Germany). We deliberately chose strains across several clades (Tarcz et al., 2013; Johri et al., 2017), mostly from the A and B clades, and one each from the C and D clades, respectively. All strains had been uninfected at the time of collection.

A mix of infected long-term cultures were used to extract parasites for the resistance assays; these cultures represented several *P. caudatum* strains [of unknown cytochrome oxidase I (COI) genotype], all different from the 30 tested strains. Infections in all cultures originated from the same parasite strain, HU1 (Dohra et al., 2013), isolated from a pond near Stuttgart (Germany) by H.-D. Görtz in 2000 and maintained in Oliver Kaltz’s lab (OK lab) since 2001.

All *P. caudatum* stocks were grown in organic lettuce medium inoculated with *Serratia marcescens* in the OK lab. Naïve and infected cultures were then transferred to Lydia Bright’s (LB; State University NY, United States) lab, and kept in wheat grass medium inoculated with *Klebsiella* (Sonneborn, 1950; Beisson et al., 2010). For extended period storage, cultures were stored at 15–18°C; amplification of cultures and resistance assays were conducted at 23°C (OK lab) or at room temperature (22–25°C; LB lab).

### *Paramecium-Holospora* Life Cycle

**FIGURE 1:**
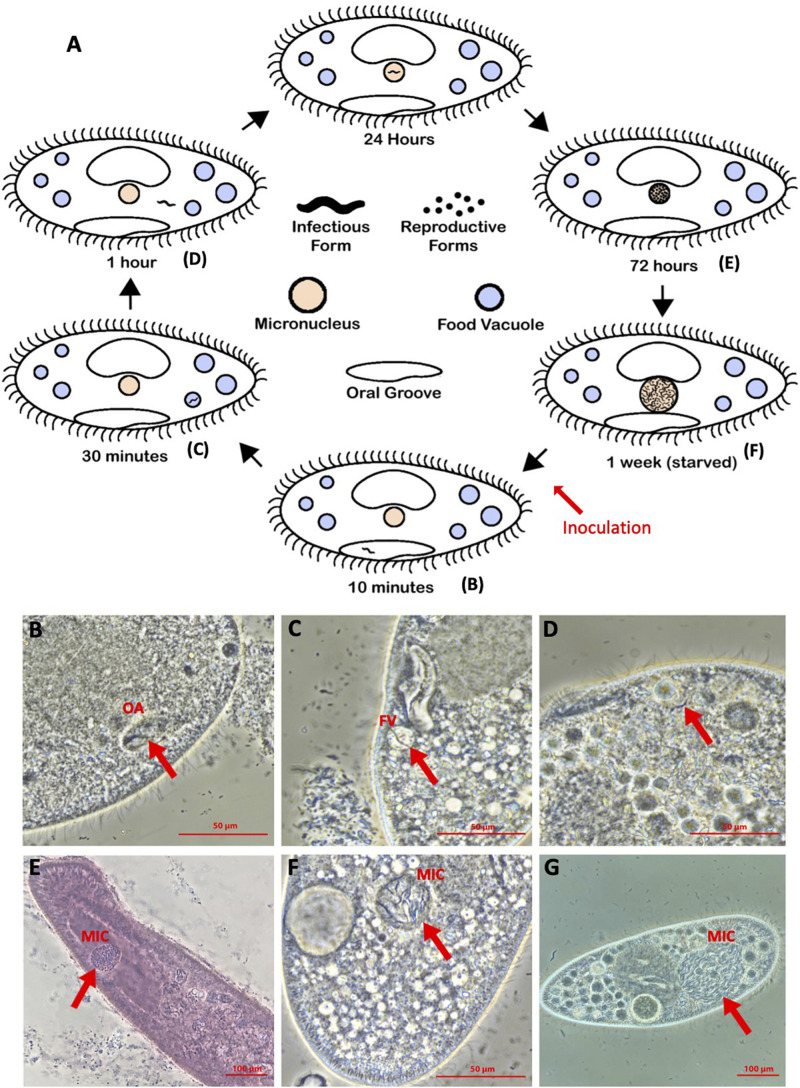
**(A)** *Holospora* life cycle, diagrammed. **(B–G)** Representative images of timepoints in infection of *Paramecium caudatum* by *Holospora undulata* (on both the diagram and the microscope image panels): **(B)** IF within the oral groove (OA) at 10 min p.i. **(C)** IF in a food vacuole (FV) at 30 min p.i. **(D)** IF in the cytoplasm at 1 h p.i. **(E)** RFs in the micronucleus (MIC) at 3 days p.i. (d.p.i.) **(F,G)** IFs and RFs in the MIC at 7 d.p.i. **(B–D,G)** Live cell images, differential interference contrast. **(E)** Cell fixed with 5% glutaraldehyde and stained with lacto-aceto-orcein. **(F)** Cells fixed with 5% glutaraldehyde. All cells imaged on a Nikon Ti-S inverted microscope, using phase contrast, 40× objective.

The infection life cycle (Figure 1) has been well-characterized for different *Holospora* species, but particularly for *H. obtusa* (Görtz and Dieckmann, 1980; Fujishima, 2009; Potekhin et al., 2018). However, the *H. undulata* life cycle has been characterized in previous studies (Görtz and Dieckmann, 1980), particularly when one considers that *H. undulata* and *Holospora elegans* appear to be different strains of the same species (Garushyants et al., 2018). Because all *Holospora* species appear to have similar life cycles, even when they infect different nuclei, we have outlined the stages, as determined in either *H. obtusa* or *H. undulata* (Figure 1A). All species show the same morphological and functional dimorphism, with IFs (elongated, up to 15 μm) and RFs (more rounded, oblong, 5 μm). A horizontally acquired infection begins when the immobile IFs are taken up by a *Paramecium* in the course of feeding through the oral apparatus, and from there reach the nascent phagosome, or food vacuole (Figures 1B,C). For successful infection, the IFs must escape the quickly acidifying food vacuole, with the aid of an electron-translucent tip (Görtz et al., 1990; Dohra and Fujishima, 1999; Fujishima, 2009) and reach the cytoplasm by 30 min to 1 h post-uptake (Figure 1D). Between 1 and 24 h post-uptake, the *H. obtusa* IF has recruited host actin in order to drive its movement across the cell (Sabaneyeva et al., 2009) to the MAC and entered the nuclear envelope. This process involves an IF-specific protein, which has two predicted actin-binding domains and translocates to the outside of the bacterial cell during invasion (Iwatani et al., 2005). The same invasion tip seems to be used to exit the food vacuole. The IFs then differentiate into the replicating RFs, thus forming characteristic clusters of bacterial cells in the nucleus. Replication continues over the first week post-infection, and the enlarging nucleus makes it easy to identify infected cells under the microscope at 400–1000× magnification (Figure 1E). After c. 1 week post-infection, a developmental switch, possibly induced when a certain bacterial cell density is reached (Fujishima et al., 1990; Kaltz and Koella, 2003; Nidelet et al., 2009), causes RFs to differentiate into IFs, thereby further enlarging the nucleus (Figures 1F,G). The IFs are released either during host cell division or upon host death, thus completing the cycle.

### Resistance Assays

#### Inoculation

Resistance assays were conducted both in the OK lab (30 strains) and in the LB lab (18 strains), in the same year (2019; Supplementary Table 1). To measure resistance, replicate cultures of a given strain, propagated clonally, were confronted with an inoculum of IFs, based on standard protocols, (e.g., Magalon et al., 2010). Briefly, large volumes of a highly infected *Paramecium* culture [up to 400 ml; preferably starved to induce the differentiation of IFs (Kaltz and Koella, 2003)], were concentrated by centrifugation, and all cells were then crushed mechanically in a bead beater to extract the IFs. The concentration of IFs was determined with a hemocytometer at 200× magnification under the microscope. In the OK lab, 10 mL samples from a given *P. caudatum* strain replicate (at carrying capacity) were gently centrifugated (700 × *g* for 15 min) and c. 1.5 mL of concentrated culture (∼3000–5000 host cells) were recovered. Then c. 10^4^ IFs from a freshly prepared inoculum were added to each replicate. Similarly, in the LB lab, 1-mL replicates of concentrated host cells (≤1.6 × 10^5^ cells) were inoculated with up to 10^5^ IFs. Final IF concentrations varied between experimental blocks (see below) but were the same for all replicates in a given block. On day 3 post inoculation (p.i.) in LB lab assays and day 4 p.i. in OK lab assays, we added 5 mL of medium to the inoculated replicates to prevent mortality due to high density. Additional medium (10–20 mL) was added after day 7 in order to maintain the replicates at carrying capacity.

#### Imaging and Infection Success

Infection success was measured by determining the number of infected Paramecia from samples of 20–30 individuals from each replicate, after lacto-aceto-orcein fixation (Görtz and Dieckmann, 1980) and inspection of the individuals at 400–1000× magnification (phase contrast). The proportion of infected cells within a sample will be referred to as infection prevalence. Infection prevalence was measured at two time points. “Early” measurements were taken on day 5 or 7 p.i., when we predicted that early differences in resistance between strains would be most apparent. Most, if not all, infections establish during the first 48 h p.i. (Fels et al., 2008); over the following days infection prevalences remain stable and RFs multiply in the micronucleus (Fels and Kaltz, 2006; Fels et al., 2008), making infected cells easy to spot under the microscope. To lend further support to this conclusion, we re-analyzed among-strain variation from the original data from Fels and Kaltz (2006) at days 2, 3, and 7, and found that infection levels differ strongly among strains, but that there was no significant day^∗^strain effect (*p* > 0.4). Therefore our early measurements represent a good quantitative estimate of host resistance, here defined as the proportion of uninfected hosts (1–infection prevalence) (Fels and Kaltz, 2006; Nidelet et al., 2009; Magalon et al., 2010).

“Late” measurements of infection prevalence were taken on day 14 (LB lab) or 21 p.i. (OK lab), when infections have taken over the micronucleus. At this point, the first infected cohort has already started to produce new secondary infections (typically during the second week p.i., (Duncan et al., 2015; Nørgaard et al., 2020) and infected hosts also show reduced division rate and increased mortality (Restif and Kaltz, 2006; Fokin and Görtz, 2009; Fujishima, 2009; Magalon et al., 2010), leading to more complex natural epidemiological dynamics in the culture (Nørgaard et al., 2020). Hence, late infection prevalences are likely determined by multiple traits, and not just host resistance.

#### Experimental Replication

For the OK lab assays, two independent experimental blocks were established 1 week apart. All replicates within a given strain were propagated clonally, through asexual divisions. For each block, three *Paramecium* replicate cultures per strain were grown to carrying capacity over 1 week prior to inoculation. One single inoculum was prepared for each block, which was then distributed over the three replicates, as described above (2 blocks × 30 strains × 3 = 180 inoculated replicates). Early measurements were taken for 177 replicates (30 strains) on day 5 p.i.; 116 late measurements (21 strains) were taken on day 21 (block 1) and day 14 (block 2). The LB lab assays (18 strains) were carried out in 4 independent blocks, over the course of 6 weeks, with a total of 38 inoculated replicates (1–4 replicates per strain; median = 2). As in the OK lab assays, independent replicate cultures and inocula were used for each block. Early measurements were taken on day 5 and 7 p.i. (67 observations) and late measurements on day 14 p.i. (38 observations). See Supplementary Table 1 for a summary of the replicates per strain and lab.

### Genotyping and Phylogeny

The relatedness of the stocks of *P. caudatum* was determined from single marker genotyping at the COI locus. Most sequences used were previously published, are deposited in GenBank under the accession numbers listed in Barth et al. (2006), and have COI genotypes listed in Supplementary Table 1. Four sequences (Hap97, 98, 99, 100) were previously unpublished but have now been deposited under the GenBank accession numbers MW183122, MW183123, MW183124, and MW183125, respectively. COI is generally considered a “neutral” marker with regard to traits under selection, such as infection phenotype in our case. DNA extractions, PCR amplification and Sanger sequencing were performed as detailed either in Barth et al. (2006) using primers COXL and COXH, or in Strüder-Kypke and Lynn (2010) using primers F199dT-B and R1143dT. An intraspecific COI phylogeny was constructed by aligning the sequences and constructing both Neighbor Joining and Maximum Likelihood phylogenies in the Geneious program^1^. Pairwise genetic distances across all strains and also within the A and B clades were calculated in Geneious and in Mega7^2^ (Kumar et al., 2016).

### Statistical Analysis

To analyze variation in infection prevalence, generalized linear mixed effect models (GLMMs) (binomial error structure, logit link) were carried out using the SAS 9.4 software (SAS Institute Inc. 2013. *SAS/STAT 13.1 User’s Guide*. Cary, NC: SAS Institute Inc.). In a first analysis, strain identity and time point (early/late) were taken as fixed effects, and laboratory (LB/OK), experimental block and experimental replicate as random factors. In a second analysis, clade (A/B) and time point were taken as fixed effects, with strain, laboratory and experimental block as random factors. Preliminary analyses indicated no significant difference between day 5 and 7 p.i. measurements in the LB lab (*p* > 0.3; correlation: *r* = 0.58, *n* = 18), and thus the data were combined as “early” estimates in the analyses.

To quantify variation in resistance among strains, we calculated the broad-sense heritability, generally defined as the fraction of genetic variation over the total phenotypic variation in a trait (Falconer, 1989). For this kind of data (clonal replicate cultures of a given strain), heritability is the intraclass correlation coefficient (ICC), i.e., the degree of similarity of resistance for replicates of the same strain. The ICC was calculated for early and late measurements of infection prevalence, across all strains combined and separately for clade A and B strains. To standardize the data, we first fitted a generalized linear model (GLM), with laboratory and experimental block as factors. On the residuals of this GLM, we performed one-way ANOVAs with strain as a random factor to obtain the variance components and to calculate the ICC (Zar, 2013) and confidence intervals (Demetrashvili et al., 2016). Finally, we also performed Mantel tests to test for correlations between pairwise genetic, geographic and phenotypic distance matrices using the “vegan” package in R (Oksanen et al., 2019). We further tested for a correlation between infection prevalence and geographical distance between host and parasite strains.

## Results

### *Paramecium caudatum* Genotyping and Geographic Origin

**FIGURE 2:**
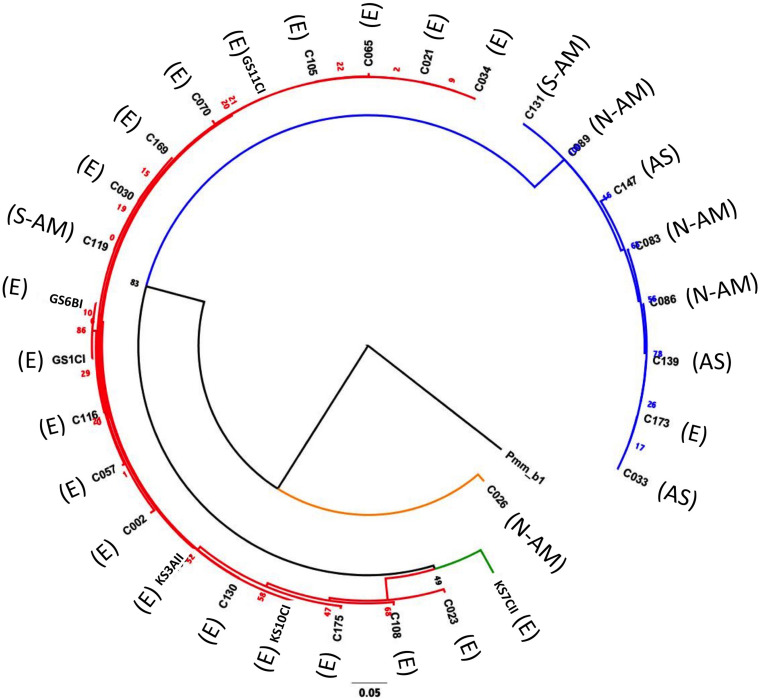
Maximum likelihood phylogeny (Tamura-Nei substitution model) constructed by comparing the COI sequence (626 nts) of the 30 *Paramecium caudatum* strains used in this study, using for an outgroup a *Paramecium multimicronucleatum* isolate PmCOI_b1_01 (accession number AM072765.1) (Barth et al., 2006). Red branches = haplogroup A; blue branches = haplogroup B; orange branches = haplogroup C; green branches = haplogroup D. Geographic origins are as follows: E = European origin; AS = Asia; N-AM = North America; S-AM = South America. The number represent maximum likelihood estimates of branch lengths (mean number of substitutions per site).

Of the 30 *P. caudatum* strains, 26 represented distinct COI sequences. Three COI sequences were shared by multiple strains. Phylogenetic analysis revealed that most strains fall into two main clades, defined by the A and B haplogroups (Figure 2 and Table 1). The average pairwise distance at the COI locus among all strains was 0.039 (i.e., 3.9% distance between strains), which is consistent with genetic distances between known reproductively isolated species in other protists (Foissner and Hawksworth, 2009). The average pairwise distances within haplogroups A and B were about four times smaller than the overall average, reflecting a clear genetic separation between the two haplogroups. Within-group levels of COI diversity were similar for the A and B groups (0.007 and 0.009, respectively). The two strains representing the C and D haplogroups were both clear outgroups to the A and B clades.

**TABLE 1:** Average pairwise genetic distance and percent identity across COI sequences (626 nucleotides) across all 30 *Paramecium caudatum* strains and within the A and B haplogroups.

	**Average pairwise distance**	**% identical sites across sequence**
All strains (30)	0.042	85.0%
Haplogroup A (20)	0.009	95.0%
Haplogroup B (8)	0.007	98.2%

*Number of strains indicated in parentheses for each calculation. Aligned using Muscle and genetic distances calculated in Mega7, using the pairwise distance function. Identical sites determined in Muscle.*

There was a clear association between haplogroup identity and geographic origin. Notably, 19 out of 20 strains from haplogroup A had been collected in Europe, while B strains were of diverse (mostly non-European) origin (Figure 2 and Supplementary Table 1). This segregation between A and B haplogroups generated an overall positive correlation between pairwise genetic distances (COI sequence differences) and geographic distances (Mantel test: *r* = 0.45, *p* < 0.0001; Supplementary Figure 2B). However, there was considerable variation around this pattern: strains with identical COI genotypes had very different geographic origins, and at a smaller scale, within Europe, the genetic-geographic correlation was weak (Mantel test: *r* = 0.15, *p* > 0.1).

### Quantitative Variation in Resistance Among Strains

**FIGURE 3:**
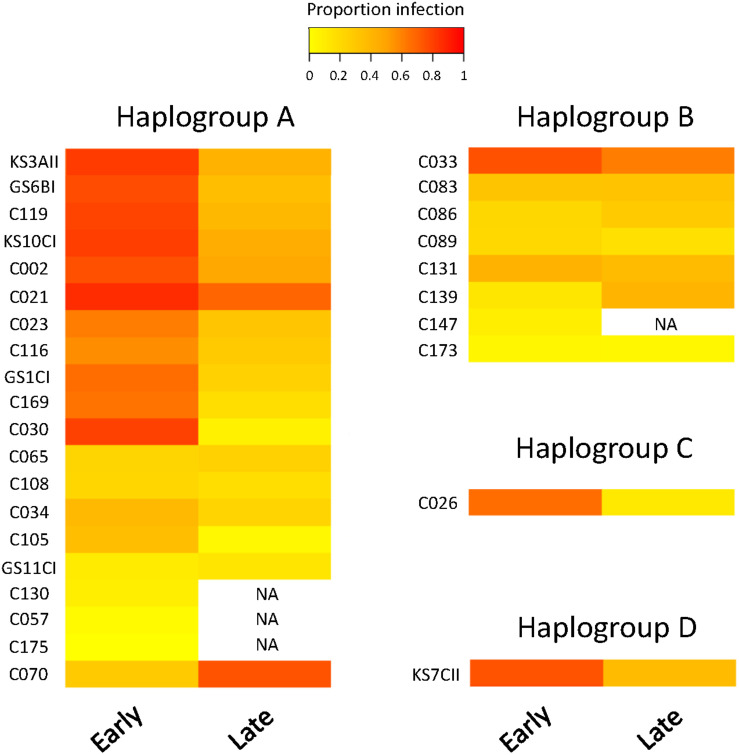
Heatmap of resistance of 30 *Paramecium caudatum* strains (from 4 COI haplogroups) against the parasite *Holospora undulata*. Measurements of infection prevalence (proportion of infected host cells) were taken 5–7 days (“early”) and 2–3 weeks (“late”) post inoculation. Each row represents a different strain. The color gradient, from yellow to red, indicates increasing levels of infection prevalence, and thus decreasing resistance.

We observed substantial variation in resistance among the 30 *P. caudatum* strains tested (Figure 3 and Supplementary Figure 1), with results being highly repeatable between laboratories (correlation of strain means: *r* = 0.75, *n* = 16, *p* = 0.0008). Variation was essentially quantitative, with only one strain (C175) showing no sign of infection in any of the inoculated replicates. For the other strains, mean levels of infection ranged from 2.8% (strain C057) to 78% (strain C021). Thus, statistical analysis revealed a significant strain effect (Table 2A) and high values of broad-sense heritability of resistance (ICC >0.5; Table 3).

**TABLE 2:** ANOVA results from GLMM models of resistance of *Paramecium caudatum* to *Holospora undulata*, as a function of strain identity, time (early vs. late measurements) and haplogroup (A vs. B).

**(A) All strains**			
*Random effects:*	Var (±SE)		
Laboratory	0.19 ± 0.24		
Experimental block (lab)	0.06 ± 0.11		
*Fixed effects:*	d.f. (n, d)	*F*	*p*
Strain	29, 298	11.7	<0.0001
Time	1, 298	57.1	<0.0001
Strain × time	25, 298	3.9	<0.0001
**(B) Haplogroup: A vs. B**			
*Random effects:*	Var (±SE)		
Laboratory	0.15 ± 0.22		
Experimental block (lab)	0.15 ± 0.13		
Strain (haplogroup)	1.70 ± 0.67		
Strain × time (haplogroup)	0.57 ± 0.21		
*Fixed effects:*	d.f. (n, d)	*F*	*p*
Haplogroup	1, 22	0.26	0.6147
Time	1, 22	5.20	0.0326
Haplogroup × time	1, 22	5.73	0.0257

*For fixed effect *F*-tests, numerator (n) and denominator (d) degrees of freedom are given. In all models, laboratory and experimental block were fitted as random factors, and their variance and standard error (SE) are given.*

**TABLE 3:** Broad-sense heritability of resistance of *Paramecium caudatum* to *Holospora undulata*, for all strains combined and for strains from haplogroups A and B, respectively.

	**Early**	**Late**
All strains	0.80 [0.69; 0.87] (30)	0.34 [0.21; 0.47] (26)
Haplogroup A	0.80 [0.57; 0.84] (20)	0.30 [0.15; 0.45] (17)
Haplogroup B	0.75 [0.45; 0.90] (8)	0.50 [0.21; 0.75] (7)

*Heritabilities represent intraclass correlation coefficients, based on measurements of infection prevalence 1 week (early) and 2–3 weeks (late) p.i. Confidence intervals (95%) shown in square brackets, the number of strains used for calculations in round brackets. Calculations based on residual infection prevalences, after correction for laboratory and block effects.*

#### Variation by Time Point

We observed a significant general decrease in infection prevalence between the early and late time points (48 ± 2% vs. 34 ± 2% SE). Although estimates of strain mean tended to be positively correlated between time points (*r* = 0.36, *n* = 26, *p* > 0.06), the significant time × strain interaction (Table 2A) indicates that differences between strains varied substantially according to time point (Figure 3). In at least two strains (C030, K3AII), there was a very strong decline in infection prevalence from initially high levels of infection to very low levels (<10%), with infection undetectable in several replicates. Although prevalence declined in the majority of strains, it remained unchanged or even increased in some cases. In the case of C070, initially low levels rose to high levels of detectable infection, although we measured only one trial for this strain. For the majority of strains, however, we found increasing variation among replicates from the early to late timepoints (Supplementary Figure 1), leading to a decline in heritability estimates by the late timepoint (Table 3). Thus, the amount of detectable among-strain variation in resistance tended to lessen over the infection time course.

#### Variation by Haplogroup

Haplogroup A strains on the whole tended to have higher levels of infection (48.9% ± 6.9 SE) than haplogroup B strains (27.3% ± 8.5 SE, Figure 3), namely at the early time point (significant haplogroup × time point interaction, Table 2B). However, both haplogroups harbor significant and similar levels of variation in this trait (Table 3). The two single strain representatives of haplogroups C and D were not included in the statistical analysis but were both highly susceptible to infection (Figure 2).

Aside from the overall differentiation between the A and B haplogroups, additional analyses provide little evidence for a link between COI genotype and resistance (Mantel test comparing genetic distances based on COI genotype and phenotypic differences in resistance: *r* = 0.01, *p* > 0.3; Supplementary Figure 2C). Indeed, strains with identical COI genotypes had widely varying resistance phenotypes (GS11CI and C105; C033, C086, C139 and C173; see Figure 3).

#### Variation by Geographic Area

**FIGURE 4:**
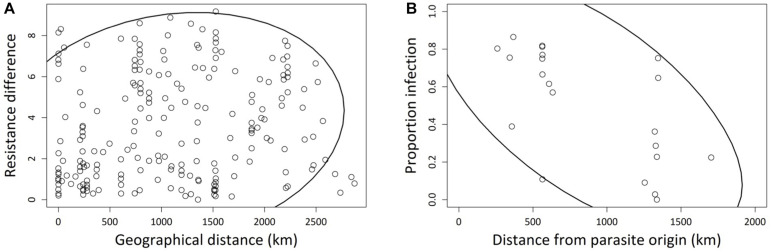
**(A)** Correlation between pairwise geographic Euclidean distances and pairwise resistance differences, for 21 European *Paramecium caudatum* strains inoculated with the parasite *Holospora undulata*. Resistance differences based on the mean residual infection prevalences for each strain, after statistically correcting for laboratory and experimental block effects. Absolute values of pairwise differences were used for the correlation analysis. Each point refers to a different pair of strains. **(B)** Correlation between infection prevalence and the geographic distance between the origin of a given *P. caudatum* strain and the origin of parasite tester isolate. Infection prevalence was averaged over multiple replicates per strain. Each point represents one strain. The 90% density ellipsoids represent a graphical indicator of the correlation between the two variables in each panel.

Although haplogroup identity coincided in part with large-scale geographic differentiation (Europe vs. rest of world; Figure 2), we found no significant overall relationship between pairwise geographic distances and differences in resistance (Mantel test: *r* = −0.02, *p* > 0.6; Supplementary Figure 2A). However, at the more regional level, within Europe, geographically closer strains tend to have more similar levels of resistance (Mantel test: *r* = 0.16, *p* = 0.0487; Figure 4A). In addition, the probability of infection of European strains was negatively correlated with their geographic distance from the site of origin of the parasite (*r* = −0.625, *p* = 0.002): strains that are geographically closer to the parasite location were more likely to become infected than those from further away (Figure 4B).

## Discussion

### Resistance Is a Quantitative Trait With Significant Variation Among Strains

Resistance is a key determinant in interactions between hosts and their parasites, and studying the amount and distribution of the variation in this trait among strains can provide insights in the possible (co)evolutionary processes in natural populations (Thompson, 2014). While previous work described qualitative patterns of resistance variation in the *Paramecium-Holospora* system, we went one step further by investigating the quantitative genetic variation in resistance to *H. undulata* across a collection of *P. caudatum* strains, using controlled inoculation experiments. Our broad-sense estimates of heritability indicate substantial levels of among-strain variation for resistance, in a pattern resembling a continuum from total resistance (0% infection prevalence) to near-complete susceptibility (80%) of strains.

From a quantitative genetics perspective, this result indicates that resistance has a strong genetic basis upon which selection can act in natural populations. Despite an artificial experimental setup, using inocula with large amounts of transmission stages, all transmission occurred naturally in our tests, and results were repeatable between laboratories and consistent over independent experimental replicate runs. Moreover, among-strain differences in resistance persisted even over multiple asexual generations in our experimental cultures, where demographic and epidemiological processes acted freely. In the longer run (several months), other studies have detected increased levels of *de novo* resistance in such cultures (e.g., Duncan et al., 2011c), demonstrating that this trait indeed responds to parasite-mediated selection.

It is important to note that we assumed in these experiments that the observed resistance phenotype of a strain is based on the germline (micronucleus) genes, somatically expressed via the macronucleus. If there were somatically acquired epigenetic mutations in the macronucleus that contributed to the resistance phenotype, these would not be propagated in the germline once conjugation occurred. Therefore, our estimates of the ICC represent the upper bound of broad-sense heritability in resistance. However, it is also important to consider that, depending on how frequent sex is in wild populations, selection may well act on this somatic genetic diversity and could thus contribute to the observed among-strain variation in resistance in wild populations. This is a typical scenario in organisms with prolonged asexual phases, such as *Daphnia* (e.g., Duncan and Little, 2007). Controlled crosses between different strains would allow us to distinguish between these two different sources of variation.

If *Holospora* truly selects for increased resistance, we need to explain why so much natural genetic variation is still present in this trait. One possibility is variation in the strength of parasite-mediated selection. Some of our strains may originate from unexposed populations and have no (co)evolutionary history with the parasite, which would explain the finding of highly susceptible strains in the collection. This is plausible, given the low incidence reported for *Holospora* infections (Fokin and Görtz, 2009). Moreover, even if *Holospora* were more frequent, high-resistance variants may not necessarily spread to fixation if resistance is associated with a cost. Indeed, trade-offs between resistance and other fitness-relevant traits are common in many host-parasite systems (Duncan et al., 2011a). For the present collection of strains, higher equilibrium density (when uninfected) tends to be associated with higher susceptibility (*r* = 0.22, *n* = 29, *p* > 0.2), indicative of a weak cost of resistance, already previously documented for our system (Lohse et al., 2006; Duncan et al., 2011a,b).

### Specificity: Evidence for Haplogroup Differences and Local Adaptation?

Another potential mechanism contributing to the maintenance of diversity among strains is genotype-specific adaptation, or more generally, host genotype × parasite genotype interactions (Lambrechts et al., 2005; Cayetano and Vorburger, 2013). Previous work indicated large-scale differences in qualitative resistance between entire syngens, or mating groups, within *Paramecium* species (Fujishima and Fujita, 1985; Rautian et al., 1993; Skoblo et al., 1996; Potekhin et al., 2018). This might reflect signatures of lineage-specific adaptation, such that certain groups of *Holospora* strains can only infect certain *Paramecium* syngens (Rautian et al., 1993). In our study, we did not find such a qualitative difference in resistance among the four highly divergent *P. caudatum* haplogroups, i.e., all haplogroups tested could be infected. However, the A and B haplogroups showed a general difference in mean resistance levels, at least at the early time point (Figure 3).

The A and B haplogroups can be broadly divided into European (A) and non-European (B) strains. We speculate that the *Holospora* strain that we used as a tester, which is of European origin, might have an evolutionary history with haplogroup-A strains and is therefore, on average, better adapted to infect strains with this same European genetic background. This is consistent with observations at a finer resolution. First, there was a weak signal of geographic distance among European strains, such that more closely located strains were more similar in their resistance phenotype (Figure 4A). This indicates that the spatial distribution of resistance follows geographic patterns of isolation by distance. Second, and more importantly, we found a negative correlation between resistance and geographic distance between parasite and host strains (Figure 4B). This distance effect likely reflects a signature of parasite local adaptation (Ebert, 1994; Kaltz and Shykoff, 1998, Kaltz et al., 1999), with infection success of our *Holospora* tester strain being highest on host strains that are geographically close to its origin (South of Germany). These interpretations are based on tests with a single parasite isolate. A thorough assessment of these patterns requires reciprocal cross-infection assays, testing parasite isolates from different haplogroups for lineage specificity, or testing sympatric and allopatric combinations of host and parasite strains for local adaptation. Such patterns readily arise in long-term experimental cultures (Nidelet et al., 2009; Adiba et al., 2010).

Infection experiments can profit immensely from accompanying molecular analysis of the geographic host (or parasite) population structure (Parratt et al., 2016; Andras et al., 2018). Our work shows that a single conserved neutral marker such as COI can be used for large-scale differentiation of lineages but is of limited utility in distinguishing closely related strains from one another. We have several instances among our strains where the COI genotypes of different strains are identical, but their infection phenotypes are divergent (Figure 3). Obviously, other loci in the genome control this trait and distinguish these different strains from one another. Future whole-genome sequencing efforts and association studies promise to untangle these genotype-phenotype interactions more definitively.

### Mechanisms of Resistance: Quantitative Variation Caused by Multiple Infection Barriers?

Our results clearly show that resistance variation is quantitative, or perhaps more precisely, probabilistic: upon inoculation, only a certain fraction of the inoculated hosts become infected. In part, this might be due to random effects related to the number of cells ingesting IFs, and thus reflect a simple dose issue. However, other studies using more than 60-fold higher dosages of IFs than in our present study also reveal substantial among-strain variation in resistance (Fels et al., 2008). Quantitative variation may thus reflect more complex mechanisms of resistance, for example if multiple host factors (and possibly interactions with multiple parasite factors) or a multi-step process (perhaps involving multiple signaling steps) determine infection success (Agrawal and Lively, 2003; Hall et al., 2019). It appears that nearly all *P. caudatum* strains examined here are potentially “colonizable” in that the defenses could be breached, allowing the parasite to enter the cell and invade the nucleus. Whether colonization occurs may depend on the efficiency of the host signaling and surveillance; this would then determine the probability of infection at the individual level and translate into a given population-level infection prevalence.

Resistance mechanisms in the host can be deployed at different points during the first steps of the infection process, including selective uptake of IFs by the host, inability of the IF to break out of the food vacuole or to enter the nucleus, or lysis in the nucleus at early stages of bacterial development (Rautian et al., 1993; Fokin and Skovorodkin, 1997; Skovorodkin et al., 2001; Fokin et al., 2005; Fels et al., 2008; Goertz, 2010). These effects should have occurred before our “early” measurement timepoint of infection prevalence. Therefore, variation at loci controlling these mechanisms could provide the quantitative genetic variation that we observed in these experiments at the early timepoint. The relative contribution of different resistance mechanisms could be investigated through repeated sampling at very early time points post inoculation, from which we could track uptake rates of IFs, the frequency and number of IFs entering the nucleus, etc. (Fels and Kaltz, 2006; Fels et al., 2008). Measurements at earlier timepoints would also reduce additional sources of variation in infection prevalence, such as differential host division rate.

Previous work has also reported strain-specific drops in infection levels or total loss of infection over longer time spans, evoking additional action of “late resistance” (Skoblo et al., 1996; Fokin and Skovorodkin, 1997; Fokin et al., 2003b). Our study confirms a general trend of decline in infection prevalence 2–3 weeks p.i., at times to very low levels (<10%) or even below detection thresholds (see Supplementary Figure 1). This trend of declining infection prevalence can be explained by several, possibly overlapping factors. Infection prevalences may drop due to incomplete vertical transmission (Restif and Kaltz, 2006; Magalon et al., 2010), and/or reduced host division and survival may not be compensated by new infections; i.e., uninfected cells outcompete infected cells over time. Variation in these parameters can be estimated from time-series data and the use of epidemiological models (Lunn et al., 2013; Nørgaard et al., 2020). However, regardless of the precise nature of the processes involved, it is important to note that among-strain differences in infection levels still persist at this later time point, despite the potential action of other traits (for which we can also expect variation to exist among strains) and the increasing noise they produce in the data.

### Conclusions and Future Directions

Our study provides a quantitative assessment of the amount and distribution of natural variation in resistance among host strains in the *Paramecium–Holospora* system and combines available information on the phylogeny and biogeography with the observed phenotypic variation. We demonstrate ample variation in resistance on which selection may act and find tentative evidence of symbiont adaptation producing signatures in geographic and/or clade-specific patterns of resistance, typical of many host-parasite systems (Thompson, 2005).

More extensive cross-inoculation tests, using both host and symbiont lines from multiple geographic locations and haplogroups or confirmed different syngens, will be required to obtain a better picture of the evolutionary forces shaping the observed variation. Such approaches can be complemented by cross mating *Paramecium* strains in standard diallel designs (Falconer, 1989) and measuring resistance in the offspring. This would eliminate potential strain-specific epigenetic or somatic variation arisen in the macronucleus (Beales and Preer, 2008) not accounted for in the present experiment. Furthermore, combining phenotypic assays with genomic studies, both whole genome sequencing and RNA sequencing over a time course, will help to identify particular gene variants involved in infection, and in so doing, point the way to the precise molecular mechanisms involved, as well as the evolutionary patterns across geography and phylogeny.

## Data Availability Statement

The datasets presented in this study can be found in online repositories. The names of the repository/repositories and accession number(s) can be found in Materials and Methods.

## Author Contributions

LB and OK conceived and designed the experiments. JW, GZ, SK, OK, NZ, LN, and WC collected the data. JW, GZ, SK, LB, and OK analyzed and interpreted the data. LB, JW, GZ, and OK drafted the manuscript. LB, GZ, LN, and OK performed the critical revision of the manuscript. All authors gave final approval of the version submitted for publication.

## Conflict of Interest

The authors declare that the research was conducted in the absence of any commercial or financial relationships that could be construed as a potential conflict of interest.

## Footnotes

1 https://www.geneious.com/home/

2 https://www.megasoftware.net

## Supplementary Material

Supplementary Figure 1 Infection prevalence in 30 *Paramecium caudatum* strains, as measured at **(A)** early time points (5/7 days) and **(B)** late time points (14/20 days) after infection with *Holospora undulata.* Light gray points are measured data, while the bigger black points are the model predictions of the statistical analysis with 95% confidence interval.

Supplementary Figure 2 Correlations between geographic Euclidean distances and resistance differences **(A)**, pairwise genetic distances (based on neutral COI marker) and geographic Euclidean distances **(B)**, and pairwise resistance differences **(C)**, for 30 *Paramecium caudatum* strains confronted with the parasite *Holospora undulata*. Resistance differences based on the mean residual infection prevalences for each strain, after statistically correcting for laboratory and experimental block effects. Absolute values of pairwise differences were used for the correlation analysis. Each point refers to a different pair of strains. The 90% density ellipsoids represent a graphical indicator of the correlation between the two variables in each panel.

Supplementary Table 1 Information on the 30 *Paramecium caudatum* strains used in this study, including geographic origin and year of sampling, cytochrome oxidase 1 (COI) genotype and haplogroup, and the number of replicates tested in the resistance assays in the Bright (LB) and Kaltz (OK) labs.
