# Supplementary figures and images for: Among-Strain Variation in Resistance of Paramecium caudatum to the Endonuclear Parasite Holospora undulata: Geographic and Lineage-Specific Patterns
Source: Front Microbiol. 2020 Dec 14;11:603046. doi: 10.3389/fmicb.2020.603046 (PMC7767928; doi:10.3389/fmicb.2020.603046)

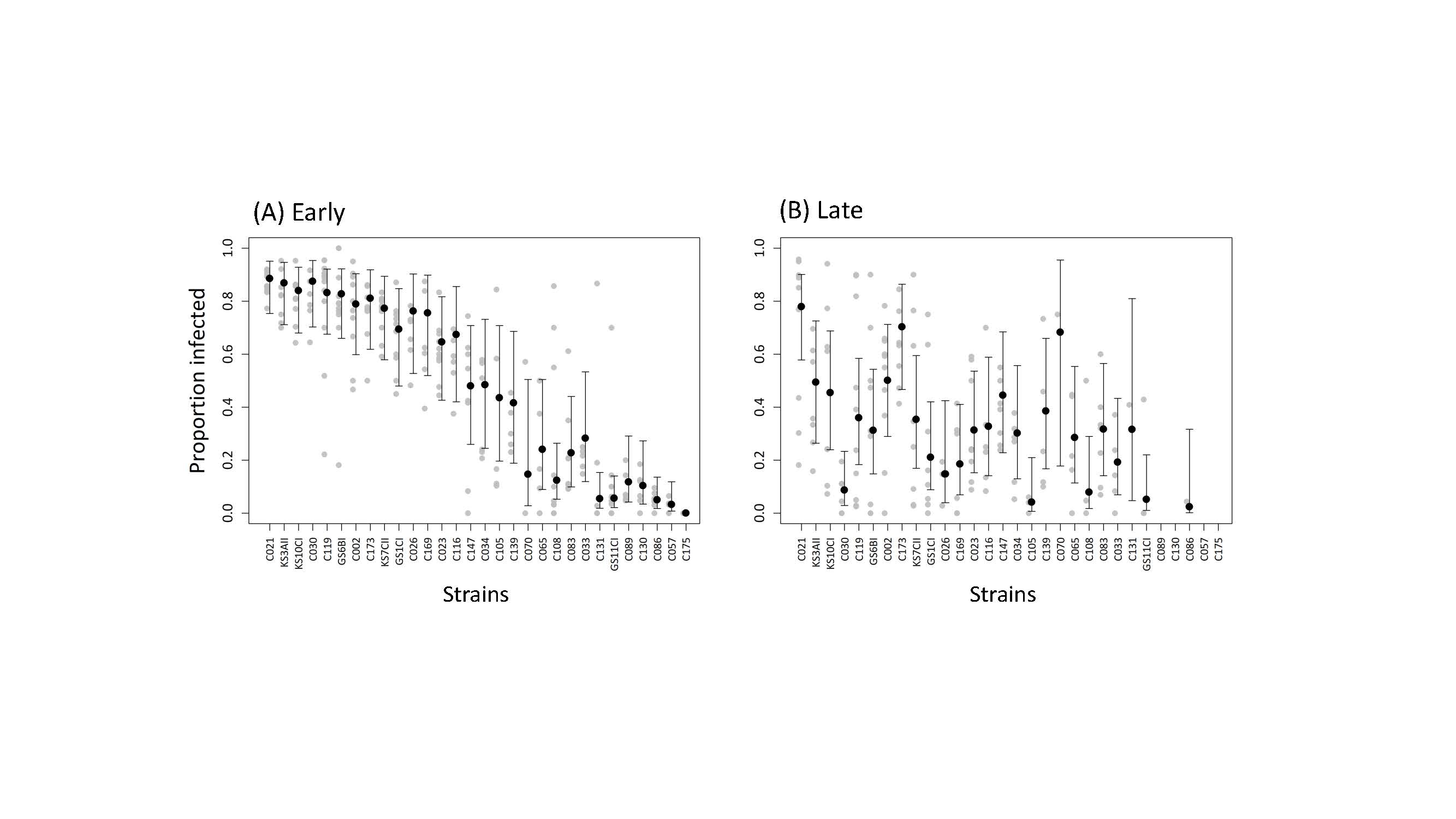

Supplement: Supplementary Figure 1 — Infection prevalence in 30 Paramecium caudatum strains, as measured at (A) early time points (5/7 days) and (B) late time points (14/20 days) after infection with Holospora undulata. Light gray points are measured data, while the bigger black points are the model predictions of the statistical analysis with 95% confidence interval. [file Image_1.jpg]

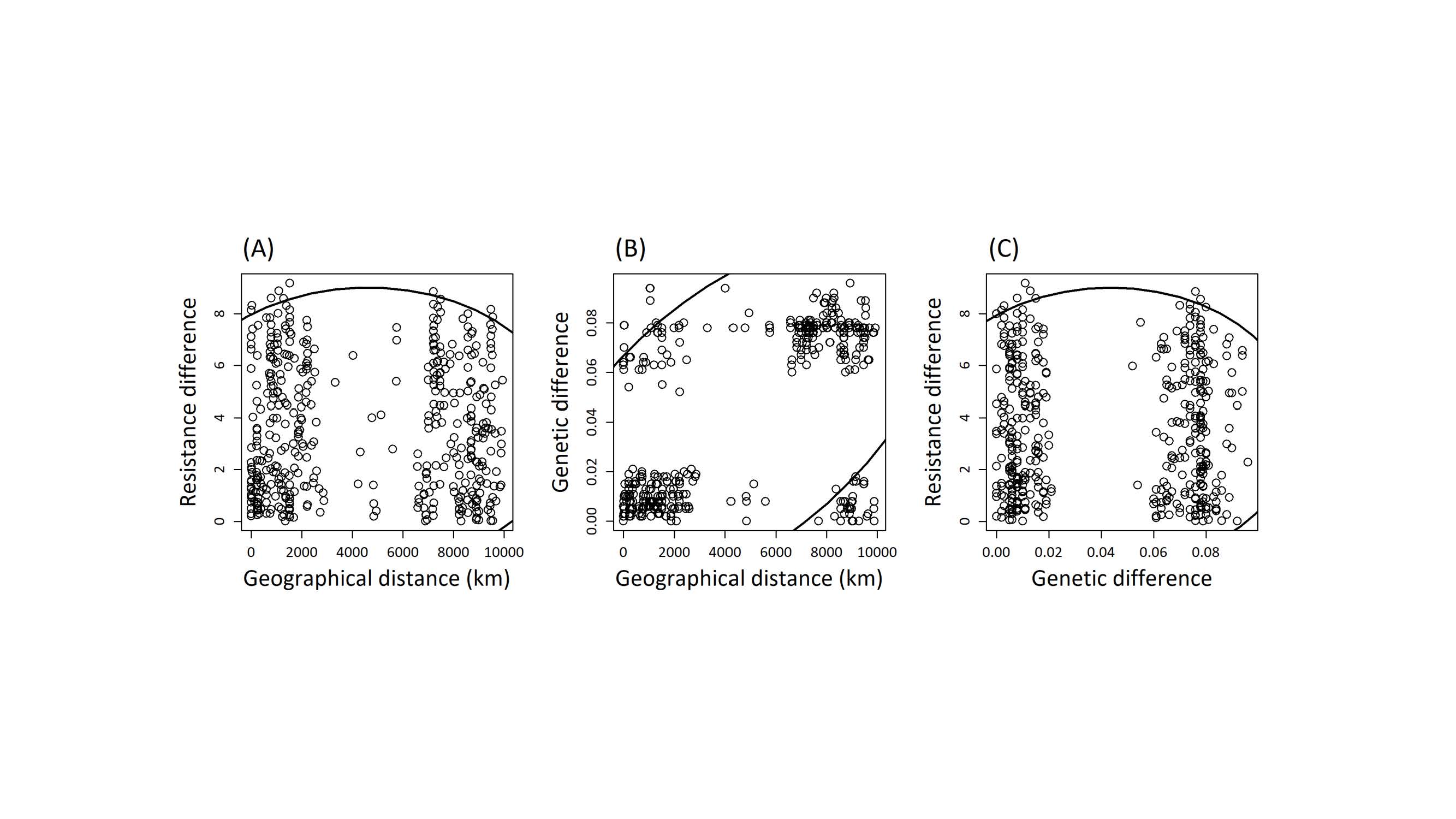

Supplement: Supplementary Figure 2 — Correlations between geographic Euclidean distances and resistance differences (A), pairwise genetic distances (based on neutral COI marker) and geographic Euclidean distances (B), and pairwise resistance differences (C), for 30 Paramecium caudatum strains confronted with the parasite Holospora undulata. Resistance differences based on the mean residual infection prevalences for each strain, after statistically correcting for laboratory and experimental block effects. Absolute values of pairwise differences were used for the correlation analysis. Each point refers to a different pair of strains. The 90% density ellipsoids represent a graphical indicator of the correlation between the two variables in each panel. [file Image_2.jpg]
